# Editorial: The roles of peripheral immune cells and their circulatory effector molecules in neuropsychiatric disorders, volume II

**DOI:** 10.3389/fncel.2026.1976330

**Published:** 2026-09-08

**Authors:** Bernhard T. Baune, Marie-Eve Tremblay, Karl Bechter, Li Tian

**Affiliations:** 1 Department of Psychiatry, University of Münster, Münster, Germany; 2 Faculty of Health, School of Medical Sciences, University of Victoria, Victoria, BC, Canada; 3 Department of Psychiatry and Psychotherapy II, Bezirkskrankenhaus Günzburg, University of Ulm, Ulm, Germany; 4 Department of Psychology, Faculty of Medicine, University of Helsinki, Helsinki, Finland

**Keywords:** immune cell, immunometabolic, inflammation, neuropsychaitric disorders, neutrophil-to-lymphocyte ratio (NLR)

Neuropsychiatric disorders are increasingly understood as multisystem conditions in which peripheral immunity, metabolic regulation, vascular interfaces, and central nervous system processes interact. Translation remains difficult, however, because inflammatory abnormalities are heterogeneous, often modest in magnitude, and shaped by a multitude of factors, including not only age, sex, lifestyle, and somatic health but also disease diagnosis, illness stage, and treatment exposure. Building on the first volume, the seven contributions to this Research Topic examine these challenges across depression, anxiety, early psychosis, and chronic schizophrenia using cellular immunophenotyping, circulating inflammatory indices, immunometabolic profiling, and mechanistic research frameworks.

Ryan et al. used flow cytometry to compare medicated patients with depression with matched healthy controls and to explore immune changes associated with electroconvulsive therapy (ECT). Reduced frequencies of CD19+ B cells and interleukin (IL)-17-producing CD8+ T cells differentiated the depression group, whereas no broad change was detected in immune cell frequencies within 72 h after ECT. Higher baseline frequencies of classical monocytes and lower frequencies of effector memory T cells were associated with greater symptomatic improvement. These findings suggest that cell-resolved immune profiles may ultimately be informative for treatment stratification, in addition to being simple diagnostic markers, while also emphasizing the necessity of larger longitudinal studies with repeated sampling across the ECT course.

Boller et al. extended cellular profiling to dendritic cells in major depressive disorder and demonstrated significant sex specificity. Severe depression was associated with reduced plasmacytoid dendritic cells and increased conventional dendritic cells. Among female individuals, IL-23-producing conventional dendritic cells were elevated and associated with symptom severity, whereas male individuals showed increases in tumor necrosis factor-, IL-1β-, and IL-6-producing dendritic cells. This study, therefore, illustrates how apparently inconsistent inflammatory findings may conceal biologically distinct immune configurations. Future studies should prospectively test whether these sex-specific profiles are reproducible, stable over time, and informative for treatment selection.

Complementing these empirical studies, Wang et al. reviewed the emerging role of T cells in depression. Their synthesis integrates evidence on T-cell trafficking across brain barriers, imbalances among regulatory and helper T-cell subsets, glial and cytokine signaling communications, brain-derived neurotrophic factor pathways, and T-cell-derived extracellular vesicles. T cells are thereby positioned not only as peripheral correlates but also as potential mediators of immune–neural communication and possible therapeutic targets. Together, the three depression-focused articles argue for a shift from undifferentiated inflammatory measurements toward functional, subset-specific, and mechanistically anchored immune assessment.

Bernal-Vega et al. examined anxiety in young adults through combined cytokine and targeted lipidomic profiling. Participants with anxiety showed higher IL-6, monocyte chemoattractant protein-1, and tumor necrosis factor-α, together with lower IL-17A, than controls. By contrast, lipid differences were modest, did not survive correction for multiple testing, and were not independently associated with symptom severity. This carefully qualified result delivers an important message: Immunometabolic signals may be present in anxiety, but the findings from small cross-sectional samples should remain hypothesis-generating rather than being interpreted as clinically deployable biomarker signatures.

The psychosis-focused contributions similarly distinguish diagnostic from state-related immune signals. Aymerich et al. found no significant difference in the neutrophil-to-lymphocyte ratio (NLR) among individuals at clinical high risk for psychosis, patients with first-episode psychosis, and healthy controls. Within first-episode psychosis, however, higher NLR was associated with more severe positive symptoms and a longer duration of untreated psychosis, while no clear cognitive associations emerged. NLR therefore appears insufficient as a stand-alone diagnostic marker but may have value as an inexpensive state marker when interpreted alongside symptom dimensions and clinical trajectories.

Shen et al. broadened the perspective to systemic and somatic manifestations in chronic schizophrenia. In a case–control study of 426 patients, splenomegaly was associated with adverse metabolic and inflammatory profiles, fatty liver, and greater negative-symptom severity. Glycated hemoglobin and negative symptoms were independently associated with splenomegaly, whereas aripiprazole use was associated with lower odds. Although the cross-sectional design precludes causal inference, the study emphasizes that immune dysregulation in schizophrenia should not be separated from metabolic diseases, medication exposure, and organ-level somatic health.

Finally, Klein et al. proposed a rigorous framework for investigating increased CD163+ perivascular macrophages reported in schizophrenia brain tissue. The framework prioritized anatomical localization, phenotypic characterization, spatial transcriptomics, single-cell or single-nucleus approaches, and appropriate psychiatric and non-psychiatric comparator groups. Infectious mechanisms and trained immunity were framed as testable possibilities rather than assumed causes. This disciplined approach provides a model for moving from provocative post-mortem observations to falsifiable, cell-specific hypotheses and for connecting peripheral immune states with cellular processes at brain interfaces.

Taken together, the Research Topic supports three overarching conclusions. First, peripheral immune abnormalities are unlikely to constitute a single transdiagnostic inflammatory signature; their meaning depends on cell type, functional state, sex, disease phase, symptom dimension, treatment, and comorbidity. Second, accessible measures such as NLR, cytokines, and routine metabolic indices may become clinically useful only when embedded in longitudinal, multimodal models rather than interpreted in isolation. Third, translation will require standardized sampling, repeated measurements, careful control of medication, body mass index, smoking, infection, circadian and lifestyle effects, and replication in independent cohorts. Where feasible, paired assessment of blood, cerebrospinal fluid, brain-interface compartments, imaging, and clinical outcomes will be essential.

Collectively, these articles advance the field from a general concept of “inflammation in psychiatry” toward a more differentiated biology of immune cell subtypes and states, immune–metabolic interactions, and brain-interface mechanisms. They also counsel appropriate restraint: Most current findings were cross-sectional and remained associative, hence requiring more rigorous prospective validation. The next phase should focus on mechanistically informed patient stratification and longitudinal intervention studies capable of determining when peripheral immune alterations are causal drivers, consequences, compensatory responses, or clinically useful indicators of neuropsychiatric illness.

